# Supporting The Mental Health Of Elite-Level Coaches Through Early Intervention

**DOI:** 10.1016/j.asmr.2023.04.017

**Published:** 2023-06-07

**Authors:** Joshua Frost, Courtney C. Walton, Rosemary Purcell, Simon M. Rice

**Affiliations:** a Centre for Youth Mental Health, The University of Melbourne, Melbourne, Australia; b Elite Sports and Mental Health, Orygen, Melbourne, Australia

## Abstract

Current evidence indicates that elite-level coaches encounter a range of performance, organizational, and personal stressors that may influence or compromise mental health. With exposure to these stressors, supports need to be established to protect and preserve the mental health of elite-level coaches. Given the paucity of evidence available, this article proposes a number of considerations that should be taken into account when developing a mental health and rehabilitation framework for high-performance coaches. We argue that early intervention should be positioned at the core of this framework, to address the onset of symptoms prior to the emergence of a mental disorder or mental health crisis. Mental health screening and monitoring of coaches, the psychological safety of high-performance environments, the mental health literacy of coaches, and the tailored pathways to support are discussed. Beyond these strategies, it is proposed that rehabilitation and reintegration should be addressed to assist coaches who are currently experiencing mental ill health or have left their role due to mental health reasons. Although further research is needed to implement evidence-based strategies, it is recommended that a future mental health framework should incorporate the perspectives of coaches to ensure it is consistent with their needs and experiences.

Coaches who operate in high-performance environments are exposed to performance, organizational, and personal stressors that have the potential to negatively impact mental health and well-being.^1^, ^2^, ^3^, ^4^, ^5^, ^6^ Elite-level coaches can be defined as individuals who operate as part of the management team or leadership group and regularly work to enhance the performance of athletes or teams competing at the Olympic, Paralympic, international, national, professional, or National Collegiate Athletic Association (NCAA) Division I level. These coaches are required to perform in rapidly evolving roles that comprise of multiple performance (strategic development, team selection, and management of assistant coaches or ancillary staff)^1^^,^^7^ and non-performance related demands (e.g., extensive travel and lack of a social life),^8^^,^^9^ all of which can lead to a lack of adequate life balance.^10^ From having to oversee the performances and well-being of athletes,^4^ to representing and personifying the public face of an organization,^7^ the highly demanding and time-consuming nature of the profession can lead to coaches spending excessive hours working and ruminating about their role.^10^^,^^11^ In addition, research also indicates that elite-level coaches should be considered as “performers” in their own right (i.e., individuals who need to manage their own psychological state, leadership style, and standard of performance to influence competitive outcomes).^1^^,^^12^ Due to these multifaceted stressors and pressures, there is growing recognition of the mental health needs of high-performance coaches and emerging empirical evidence indicating that elite-level coaches may be susceptible to mental ill health.^13^, ^14^, ^15^

## Conceptualizing Mental Health in High-Performance Sport

Researchers have used a variety of definitions and frameworks to operationalize mental health throughout the high-performance discourse.^16^ One of the leading theoretical approaches is Keyes’ dual-continua model of mental health,^17^^,^^18^ which asserts that mental health comprises two distinct but related phenomena: (i) the presence or absence of well-being (e.g., emotional, psychological, and social) and (ii) the presence or absence of a mental disorder (i.e., mental ill health). The model postulates that well-being refers to one’s perceived quality of life, and reflects an individual’s evaluation of their emotional (e.g., positive affect), psychological (e.g., personal growth), and social (e.g., contributions to the community, workplace, and society) well-being.^19^ When combined, these dimensions determine whether an individual is flourishing (high well-being), experiencing moderate mental health, or languishing (low well-being). Mental ill health, on the other hand, refers to the various symptoms and disorders associated with mental illness. This spectrum can be used to assess whether an individual is absent of symptoms or experiencing mild to severe symptoms of mental ill health. Given that this framework has been empirically supported in elite, university, and youth sport settings,^20^, ^21^, ^22^ we argue that, to understand an individual’s complete state of mental health, both symptoms associated with well-being and mental ill health should be examined.

Although the domains of well-being and mental ill health may overlap, an individual experiencing low well-being may not necessarily meet diagnostic criteria for a mental disorder. Conversely, those with a diagnosed mental disorder could potentially experience high levels of well-being throughout their day-to-day lives. Keyes’ model is beneficial to researchers and practitioners because it provides a conceptual framework that can be used to categorize and distinguish between an individual experiencing low well-being or mental ill health (or both). Since research indicates that individuals who “languish” for prolonged periods are more susceptible to developing mental disorders over time,^23^, ^24^, ^25^ this emphasizes the importance of providing early, timely interventions to halt the progression and development of mental disorders in elite-level coaches. Given the proliferation of research into the mental health of elite athletes^26^^,^^27^ and the numerous mental health position statements that have emerged from this research,^28^, ^29^, ^30^, ^31^ it is critical that the field expand to develop frameworks that consider the mental health needs and challenges faced by elite-level coaches.

## Mental Ill Health in Elite-Level Coaches

Despite scant research to date, preliminary evidence suggests that elite-level coaches are potentially vulnerable to a range of mental disorders. Findings indicate that high-performance coaches experience symptoms associated with depression,^9^^,^^10^^,^^14^^,^^15^^,^^32^^,^^33^ anxiety,^9^^,^^10^^,^^14^^,^^15^^,^^34^ gambling disorders,^35^ alcohol and substance abuse,^14^^,^^15^^,^^33^ and eating disorders.^15^^,^^36^ Preliminary evidence indicates that rates of mental ill health may range from 14% to 44% for depression,^9^^,^^14^^,^^32^ 40% to 44% for anxiety,^9^^,^^14^ and 19% to 48% for alcohol abuse^9^^,^^14^ in elite-level coaches (i.e., presence of symptoms). In addition to this, a range of studies have also identified several risk factors that may contribute toward mental ill health. For instance, having a family history of a mood disorder (depression),^32^ managing an extensive list of stressors (anxiety and depression),^9^^,^^32^ adopting ineffective coping strategies (anxiety),^34^ coaching a team sport (adverse alcohol use),^9^ and experiencing stigma towards help-seeking^33^ (adverse alcohol use and depression) have been found to be associated with symptoms of mental ill health in elite-level coaches. Although further research is needed to confirm the prevalence and associated risk factors with larger cross-national samples (e.g., n >100),^26^ these initial findings suggest that high-performance coaches may be at a greater risk of developing mental disorders when compared with elite athletes.^27^

### The Need for Early Intervention

One of the most effective ways to provide timely support to coaches is through early intervention. This strategy has been recommended by researchers for both elite and youth elite athletes.^37^^,^^38^ This stage of intervention reflects a period where the initial onset and presentation of symptoms has occurred, but the development (and sometimes diagnosis) of a clinical disorder is considered to be premature.^39^ Given the importance that timing plays in managing the exacerbation of symptoms, this period is perceived to be critical to suppressing the progression and maturation of a mental disorder.^40^ Evidence indicates that providing care or treatment before the onset of a mental disorder can result in improved outcomes when compared with treatment as usual responses (e.g., reduced severity of symptoms).^41^, ^42^, ^43^, ^44^, ^45^ Since there is a growing body of evidence to suggest that elite-level coaches may experience high levels of stress or burnout at some point throughout their career,^7^^,^^46^, ^47^, ^48^ approaches that seek to halt the development of a mental disorder or mental health crisis should be carefully considered. As a result, this article will discuss a number of factors that should be addressed when developing an early intervention framework for elite-level coaches. In adding to these, we propose strategies to support the recovery and continued care for coaches who are currently experiencing or have previously experienced mental ill health. This includes avenues to reintegrate individuals who have departed the coaching profession due to mental health reasons.

### Early Interventions in Elite Sport

In an elite sports context, Purcell and colleagues^37^ proposed an early intervention framework to support and respond to the mental health of elite athletes. The framework discusses four phases of involvement, including prevention, athletes identified as being “at risk” of mental ill health, early intervention for first episodes of illness, and specialist mental health care. Each stage incorporates a variety of strategies that can be used to assist elite athletes with diverse experiences of mental health. For instance, the prevention stage proposes that mental health literacy courses, individual development programs, and mental health screening and feedback can be utilized to support athletes prior to the onset of symptoms, whilst more specialized practitioners and treatments are required for acute and complex presentations. This framework also promotes and emphasizes the importance of considering aspects of the broader sporting “ecology” in the development or maintenance of mental ill health, including factors at the interpersonal level (e.g., team dynamics), organizational level (e.g., leadership or cultural practices within the club or code), and wider societal level (e.g., public scrutiny or social media abuse). This framework was developed to promote better support for the mental health of elite athletes, but may also hold relevance to elite-level coaches, particularly in relation to minimizing the myriad stressors that potentially operate as risk factors for mental ill health (e.g., lack of role clarity, job insecurity),^9^ and promoting early detection and intervention (e.g., encouraging mental health help-seeking)^33^ so that mental health crises are averted.

## Early Intervention for Mental Health In Elite-Level Coaches

### Mental Health Screening and Monitoring

To identify coaches who may be at risk or currently experiencing a mental disorder, screening tools are an effective measure that can be employed to detect symptoms associated with mental ill health. In 2021, the International Olympic Committee developed the Sport Mental Health Assessment Tool 1 (SMHAT-1) and Sport Mental Health Recognition Tool 1 (SMHRT-1) to examine symptoms of mental ill health in elite athletes.^49^ These tools were designed to provide mental health practitioners and members of an athlete’s entourage (e.g., friends and families) with a set of guidelines that can identify and treat athletes who may be experiencing symptoms of mental ill health. The SMHAT-1, for example, uses the Athlete Psychological Strain Questionnaire (APSQ) to measure symptoms of athlete-specific distress. The APSQ evaluates an athlete’s ability to self-regulate, capacity to perform, and proclivity to implement maladaptive external coping mechanisms to reflect and measure an athlete’s level of psychological strain.^50^^,^^51^ APSQ items are not necessarily relevant to coaches, and a need remains to develop and validate measures that consider the unique stressors and symptoms experienced by this cohort. Although the Coach Burnout Questionnaire^52^ and Burnout Prevention Questionnaire for Coaches^53^ have been generated to assess symptoms associated with burnout, it has been argued that these measures possess conceptual weaknesses (e.g., theoretical and clinical limitations).^54^ Academics and industry stakeholders would subsequently benefit from a robust tool that identifies high-performance coaches at risk of psychological distress and mental ill health. A tool of this nature could assist with early identification and provide opportunities to intervene before the development of a mental disorder or mental health crisis. Measures incorporated to assess disorder-specific symptoms within the SMHAT-1 may be a viable alternative during the interim (e.g., the GAD-7 for symptoms of generalized anxiety; PHQ-9 for symptoms of major depression).

Given the current sensitivity of disclosing mental health concerns in high-performance sport, screening processes should be ethically managed, ideally by qualified mental health practitioners or sports medicine physicians.^50^ Since coaching in elite sports is often accompanied by high levels of job insecurity and scrutiny,^55^^,^^56^ coaches may be wary about potentially sensitive mental health information being shared with others in (and beyond) the organization, including those occupying board or management positions, given the potential implications toward their employment status and how they are perceived within the sport. For mental health screening to be effective as an early intervention strategy, coaches need to feel assured about their confidentiality and the ethical premise for screening (i.e., early detection and treatment for mental health concerns, rather than “screening out”).

During the screening process, it is vital that timing is taken into consideration when assessing the mental health of high-performance coaches. Although screening can be delivered during standard competitive periods (e.g., preseason and midseason),^57^ organizations and federations should also take specific periods when coaches may be at greater risk of mental ill health into account.^30^ For instance, coaches who are approaching or have recently experienced retirement may be at a heightened risk of developing symptoms associated with depression.^32^ Similarly, coaches who have been recently dismissed may also face periods of distress and uncertainty.^58^ Organizations and federations should be mindful of the various transitional phases faced by coaches, and that their duty of care should also extend beyond a coach’s tenure. This may contribute toward coaches feeling a greater sense of organizational support, which may increase the likelihood of a coach returning to the role, in the event that they step away to manage an episode of mental ill health.

### Mental Health Literacy

The beliefs and attitudes about mental health can play a pivotal role in early identification and responses to the initial onset of symptoms.^59^ It is critical that an early-intervention framework considers and promotes mental health literacy in elite-level coaches. Given that there is some evidence to suggest that coaches possess lower levels of mental health literacy than other populations (e.g., university students and health care professionals),^60^^,^^61^ educational programs need to be established and tailored to accommodate for the specific stressors and symptoms experienced by coaches. Despite the value of these findings, to the best of our knowledge, no research has explored the mental health literacy of elite-level coaches exclusively. We therefore argue that research is urgently needed to better understand how high-performance coaches perceive mental health, examining their knowledge and beliefs in relation to this. Not only is this important for coaches themselves, but given that coaches operate within an athlete’s interpersonal network and have been identified as the potential “gatekeepers” to mental health supports,^62^, ^63^, ^64^, ^65^ attitudes that promote mental health and help-seeking can positively influence the management and perceptions of an athlete’s mental health.

Although interventions have been developed to enhance the mental health literacy of coaches,^66^^,^^67^ the focus has largely been directed toward providing support for their athletes. As a result, there is a need for interventions to focus on the mental health of coaches to ensure that these individuals are cognizant of their symptoms and behaviors, in conjunction with an awareness of when support may be required. Current mindfulness-based interventions have shown early promise in cultivating greater emotional awareness in high-performance coaches.^68^^,^^69^ Given these studies found that greater self-awareness led to improved mental well-being, mindfulness-based interventions could be incorporated as a potential strategy to enhance the awareness and identification of a coach’s emotional state and mental health. Before the implementation of these strategies, however, further research is needed to confirm these findings with larger sample sizes and robust theoretical approaches.

### Psychological Safety

Though initially developed as a construct outside of sport, over recent years psychological safety has garnered increased attention in sporting contexts.^70^ Although there are major concerns about the conceptual clarity and application of this term, Vella et al.^70^ recently provided a working definition of psychological safety in sport as “the perception that one is protected from, or unlikely to be at risk of, psychological harm in sport.” Cultivating psychologically safe environments may encourage elite sportspeople to take greater interpersonal risks given there are fewer consequences attached to one’s individual actions (e.g., raising issues or sharing sensitive information).^71^ Preliminary evidence indicates that psychologically safe environments may contribute toward improved mental health (e.g., reduced anxiety) and performance-related outcomes (e.g., team effectiveness) in sport.^70^ This has been qualitatively supported in a coaching context, where it has been found that Olympic coaches who were provided with the latitude to make mistakes reported reduced stress.^8^

Given that psychologically safe climates might empower individuals to disclose mental health problems and pursue mental health services, an early intervention framework should also consider the environmental supports that could be established to protect the mental health of elite-level coaches. Recently, Rice and colleagues^72^ developed a psychometric tool to measure the perceived psychological safety of an elite sports environment. The Sport Psychological Safety Inventory (SPSI) utilizes three dimensions to examine psychological safety, including factors associated with mentally healthy environments, mental health literacy, and mental health stigma. This instrument may offer value to a prospective mental health framework, as the SPSI was validated with a sample incorporating elite sports coaches and high-performance staff. We therefore propose that organizations could use the SPSI to examine the perceived safety of a coach’s high-performance environment. This assessment could be utilized to identify whether sporting organizations need to better support their coaches, in order to cultivate environments that encourage coaches to prioritize and manage their mental health.

### Pathways to Mental Health Supports

When developing a mental health framework for high-performance coaches, pathways to mental health supports should also be considered. Coaches are prone to symptoms associated with stress and burnout,^7^^,^^47^^,^^73^^,^^74^ which, if ignored or otherwise neglected, could develop into more serious and persistent mental health symptoms or disorders. The provision of psychological or mental health services (e.g., by psychologists or psychiatrists) may play a crucial role in thwarting the exacerbation of symptoms. For instance, it has been found that help-seeking behaviors were positively correlated with levels of well-being in a sample of British coaches from a range of performance levels.^60^ One of the greatest threats to accessing mental health supports in elite sport, however, arises from stigma.^75^ Initial research suggests that some high-performance coaches may experience stigma towards mental health help-seeking,^33^ with evidence indicating that coaches may avoid mental health services in the attempt to suppress and conceal what they perceive to be signs of vulnerability and weakness.^10^ Coaches may neglect treatment because of the common misperception that help-seeking is incongruous with the ideals of stoicism and mental toughness—misunderstood values that are commonly promoted and glorified in elite sport.^76^^,^^77^ To better support coaches, sporting organizations, federations, and governing bodies need to nurture high-performance cultures that destigmatize mental ill health and reposition help-seeking. Future research might examine the relationship between mental health and coaching effectiveness to leverage proponents of positive psychology, and evaluate whether performance could be employed as a “hook” to engage elite-level coaches with mental health practices and help-seeking.

Despite the presence of stigma, there may be contexts in which elite-level coaches seek to engage with psychological services.^15^ For example, Pilkington and colleagues^14^ found that 35% of an Australian elite-level coaching sample had previously sought psychological treatment at some stage throughout their life. Although based on a limited sample size (n = 78), given that more than one third of coaches reported seeking psychological treatment, this finding indicates a potential receptiveness toward mental health services. This may especially be the case in which mental health services are facilitated and paid for by the sporting body, as in the Mental Health Referral network funded by the Australian Institute of Sport,^78^ and available to both high-performance athletes and coaches. As a result, we argue that further research is needed to understand the range of barriers and facilitators that influence mental health help-seeking in elite-level coaches. For instance, education and accessibility have been found to act as key barriers for elite athletes.^75^ Although there may a considerable overlap among athletes, coaches, and high-performance support staff, factors such as job security may play a larger role in a coach’s consideration of support due to the threat and frequency of coach turnover.^79^ Researchers should subsequently seek to engage with coaches to understand their preferences for mental health help-seeking (e.g., how and with whom), and ideally co-design mental health services or frameworks to meet their specific needs.

## Rehabilitation and Reintegration

Although it is critical to intervene at the earliest opportunity when symptoms of mental ill health arise, it is neither realistic nor appropriate to completely *prevent* experiences of mental ill health. Coaches—like other members of the community—will be susceptible to episodes of psychological distress from general life adversity (such as relationship or financial difficulties or illness or death of loved ones), in addition to work-related challenges and setbacks. Evidence-based psychological and pharmacological treatments exist for a range of mental disorders^30^ and should be considered for elite-level coaches in accordance with clinical guidelines. Contextual issues (e.g., personal, social, environmental, and psychological) should also be factored into such treatments for coaches.^80^ For instance, to manage stressors, research suggests that high-performance coaches may be prone to using nonadaptive coping mechanisms such as alcohol consumption and substance use.^4^^,^^9^^,^^33^^,^^81^ Preliminary evidence indicates that elite-level coaches may engage in risky alcohol practices and substance abuse more than athletes and high-performance support staff.^14^^,^^15^ Exploring treatments that promote adaptive coping could subsequently be a fruitful area for further research. These treatments should also consider whether a coach is still operating within their role or is no longer employed. This article stresses that the duty of care for coaches needs to be extended beyond their tenure, given the risks associated with transitional phases (e.g., retirement and dismissals).^32^^,^^56^

Since Keyes’ model asserts that an individual can flourish (e.g., successfully achieving performance outcomes) despite the diagnosis of a mental disorder, we argue that sporting organizations must prioritize support and early intervention over dismissal or prolonged stand-downs for coaches experiencing an episode of mental ill health. This support is paramount if the high-performance sports industry wishes to maintain the best-in-class coaches. If elite-level coaches are fearful or apprehensive about the implications of a mental health crisis or disorder, stigma will continue to persist, and the desirability of the role may decrease, given there may be more stable, supportive, and appealing opportunities elsewhere. To ensure high-quality leaders stay within the industry, coaches need to feel supported by their relevant organizations or federations, rather than isolated or detached.

For elite-level coaches who experience mental ill health, some individuals may feel it necessary to depart from the role.^10^^,^^33^^,^^82^ Evidence indicates that sufficient psychological recovery (e.g., psychological detachment and relaxation)^48^^,^^83^^,^^84^ and taking time away from the high-performance environment^10^^,^^82^ can lead to improved mental health outcomes in elite-level coaches. For those who take periods of leave for mental health reasons, however, appropriate protocols need to be established to ensure coaches return to psychologically healthy environments where they can perform their roles without being at risk of relapse if any contributing factors still persist. Currie and Purcell^85^ have suggested that return-to-play guidelines adopting a traffic light system (e.g., red, amber, green ratings) representative of an individual’s clinical condition would be a welcomed addition to the elite sports industry. These guidelines could emulate protocols put in place to assist athletes returning from significant physical injuries (e.g., concussion).^37^ Given that this is a broader area that could help generate parity between physical and mental health in elite sports environments, it is important that coaches remain part of the rehabilitation conversations and processes.

## Conclusion

There is growing recognition that elite-level coaches, who operate in the same high-performance and outcome-focused environments as athletes, might also be susceptible to the same mental health difficulties their athletes face. Empirical research evaluating the mental health of elite-level coaches, however, is still in its infancy. This article has subsequently proposed a range of factors that should be considered when developing a framework to support the mental health of elite-level coaches (Table 1). Although these measures reflect the distinct stressors experienced by coaches, as well as the unique contexts they operate within, a greater body of evidence is needed to inform and develop a prospective framework. At present, little is known about the extent to which elite-level coaches experience mental ill health, as well as the various risk and protective factors that operate at the individual, interpersonal, organizational and societal levels. Current research also generally reflects the mental health experiences of coaches who identify as men. Much like the broader representation of gender in the field of sports psychology,^86^ future research is required to explicitly consider gender differences and potentially other forms of diversity, including race and disability (e.g., para-coaches). For instance, given the under-representation of women coaches within elite sport,^87^ further research should evaluate the gender-specific stressors and risk factors that may influence the mental health of coaches who identify as women (e.g., managing hypermasculine cultures).^88^

**Table 1:** Early Intervention Recommendations

Early Intervention	Mental Health Screening	Mental Health Literacy	Psychological Safety	Mental Health Support
Purpose	To identify mental health symptoms and disorders in elite-level coaches.	To understand the beliefs and attitudes that elite-level coaches hold toward mental health and help-seeking.	To cultivate high-performance environments that protect coaches from mental ill health, support openness and connection, and encourage individuals to seek mental health services.	To provide elite-level coaches with pathways and access to evidence-based mental health supports and services.
Recommendations	In the absence of a coach-specific mental health screening tool, the SMHAT-1 could be used as a guide to assess general mental health symptoms and disorders in elite-level coaches (e.g., anxiety and depression).	Given mental health literacy has not been studied exclusively in elite-level coaches, research is needed to better understand coaches’ beliefs and attitudes toward mental health and help-seeking. Mental health literacy levels could be examined using the Mental Health Literacy Survey.^89^	Future research should examine how elite-level coach’s perceive psychological safety in their high-performance environments. The SPSI could be used as a measure to assess and benchmark psychological safety in coaching cohorts.	Since preliminary evidence indicates there may be an appetite for elite-level coaches to engage with psychological services, tailored referral pathways and mental health services should seek to optimize coach-specific support (e.g., how and with whom?)

To develop a mental health framework that could provide coaches, practitioners and organizations with a set of guidelines to manage coach mental health, researchers need to better understand how elite-level coaches experience mental health. Researchers should also investigate theory-based strategies and interventions that could be employed to protect coaches from mental ill health (e.g., compassion-focused approaches are relevant in sport and currently being trialed with coaches).^68^^,^^89^^,^^90^ Alongside evidence-based practices, future mental health frameworks should incorporate co-design processes that ensure the mental health help-seeking and intervention preferences of coaches are considered and incorporated. Without such knowledge and engagement, mental health services or frameworks may not be “fit for purpose” and therefore risk redundancy due to a lack of potential relevancy and suitability.

Beyond the contents of a framework, timing is also a critical aspect to consider. Elite-level coaches are not immune to high levels of stress and periods of burnout, which necessitates the need for early, timely interventions to prevent the onset of mental ill health, or at least the worsening of existing symptoms. For those who do experience mental ill health, supports and treatment plans need to be established, so that coaches are provided opportunities to recover within their role. It is also incumbent upon sports organizations to offer rehabilitative support after a coach departs from their role. From a sustainability perspective, offering psychological support to an individual even if they are no longer coaching, may potentially have lasting impacts in retaining these “bastions of leadership” within the high-performance environment. A mental health framework for elite-level coaches should subsequently address ways to support coaches beyond their tenure.

## Supplementary Data

ICMJE author disclosure forms

## References

[1] Thelwell R.C., Weston N.J.V., Greenlees I.A., Hutchings N.V. (2008). Stressors in elite sport: A coach perspective. J Sports Sci.

[2] Olusoga P., Butt J., Hays K., Maynard I. (2009). Stress in elite sports coaching: Identifying stressors. J Appl Sport Psychol.

[3] Baldock L., Cropley B., Neil R., Mellalieu S.D. (2021). Stress and mental well-being experiences of professional football coaches. Sport Psychol.

[4] Didymus F.F. (2017). Olympic and international level sports coaches’ experiences of stressors, appraisals, and coping. Qual Res Sport Exerc Health.

[5] Levy A., Nicholls A., Marchant D., Polman R. (2009). Organisational stressors, coping, and coping effectiveness: A longitudinal study with an elite coach. Int J Sports Sci Coaching.

[6] Rhind D.J., Scott M., Fletcher D. (2013). Organizational stress in professional soccer coaches. Int J Sport Psychol.

[7] Knights S., Ruddock-Hudson M. (2016). Experiences of occupational stress and social support in Australian Football League senior coaches. Int J Sports Sci Coaching.

[8] Chroni S.A., Abrahamsen F., Hemmestad L. (2016). To be the eye within the storm, I am challenged not stressed. J Appl Sport Psychol.

[9] Kegelaers J., Wylleman P., van Bree I.N.A., Wessels F., Oudejans R.R.D. (2021). Mental health in elite-level coaches: Prevalence rates and associated impact of coach stressors and psychological resilience. Int Sport Coaching J.

[10] Olusoga P., Kenttä G. (2017). Desperate to quit: A narrative analysis of burnout and recovery in high-performance sports coaching. Sport Psychol.

[11] Frey M. (2007). College coaches’ experiences with stress—“Problem solvers” have problems, too. Sport Psychol.

[12] Gould D., Greenleaf C., Guinan D., Chung Y. (2002). A survey of U.S. Olympic coaches: Variables perceived to have influenced athlete performances and coach effectiveness. Sport Psychol.

[13] Smith A., Haycock D., Jones J., Greenough K., Wilcock R., Braid I. (2020). Exploring mental health and illness in the UK sports coaching workforce. Int J Environ Res Public Health.

[14] Pilkington V., Rice S.M., Walton C.C. (2022). Prevalence and correlates of mental health symptoms and well-being among elite sport coaches and high-performance support staff. Sports Med Open.

[15] Åkesdotter C., Kenttä G., Eloranta S., Håkansson A., Franck J. (2022). Prevalence and comorbidity of psychiatric disorders among treatment-seeking elite athletes and high-performance coaches. BMJ Open Sport Exerc Med.

[16] Lundqvist C., Andersson G. (2021). Let's talk about mental health and mental disorders in elite sports: A narrative review of theoretical perspectives. Review. Front Psychol.

[17] Keyes C. (2002). The mental health continuum: From languishing to flourishing in life. J Health Soc Behav.

[18] Keyes C. (2005). Mental illness and/or mental health? Investigating axioms of the complete state model of health. J Consult Clin Psychol.

[19] Keyes C.L.M., Bauer G.F., Hämmig O. (2014). Bridging Occupational, Organizational and Public Health: A Transdisciplinary Approach.

[20] Kuettel A., Pedersen A.K., Larsen C.H. (2021). To Flourish or Languish, that is the question: Exploring the mental health profiles of Danish elite athletes. Psychol Sport Exerc.

[21] Van Slingerland K.J., Durand-Bush N., Rathwell S. (2018). Levels and prevalence of mental health functioning in Canadian university student-athletes. Can J Higher Educ.

[22] Kuettel A., Durand-Bush N., Larsen C.H. (2022). Mental health profiles of Danish youth soccer players: The influence of gender and career development. J Clin Sport Psychol.

[23] Keyes C.L.M., Dhingra S.S., Simoes E.J. (2010). Change in level of positive mental health as a predictor of future risk of mental illness. Am J Public Health.

[24] Wood A.M., Joseph S. (2010). The absence of positive psychological (eudemonic) well-being as a risk factor for depression: A ten year cohort study. J Affect Disord.

[25] Grant F., Guille C., Sen S. (2013). Well-being and the risk of depression under stress. PLoS ONE.

[26] Rice S.M., Purcell R., De Silva S., Mawren D., McGorry P.D., Parker A.G. (2016). The mental health of elite athletes: A narrative systematic review. Sports Med.

[27] Gouttebarge V., Castaldelli-Maia J.M., Gorczynski P. (2019). Occurrence of mental health symptoms and disorders in current and former elite athletes: A systematic review and meta-analysis. Br J Sports Med.

[28] Moesch K., Kenttä G., Kleinert J., Quignon-Fleuret C., Cecil S., Bertollo M. (2018). FEPSAC position statement: Mental health disorders in elite athletes and models of service provision. Psychol Sport Exerc.

[29] Henriksen K., Schinke R., McCann S. (2020). Athlete mental health in the Olympic/Paralympic quadrennium: a multi-societal consensus statement. Int J Sport Exerc Psychol.

[30] Reardon C.L., Hainline B., Aron C.M. (2019). Mental health in elite athletes: International Olympic Committee consensus statement (2019). Br J Sports Med.

[31] Van Slingerland K.J., Durand-Bush N., Bradley L. (2019). Canadian Centre for Mental Health and Sport (CCMHS) position statement: Principles of mental health in competitive and high-performance sport. Clin J Sport Med.

[32] Kim S.S.Y., Hamiliton B., Beable S., Cavadino A., Fulcher M.L. (2020). Elite coaches have a similar prevalence of depressive symptoms to the general population and lower rates than elite athletes. BMJ Open Sport Exerc Med.

[33] Roberts S.J., Baker M., Reeves M.J., Jones G., Cronin C. (2019). Lifting the veil of depression and alcoholism in sport coaching: how do we care for carers?. Qual Res Sport Exerc Health.

[34] Lee Y.H. (2021). The roles of different appraisals in anxiety and emotional exhaustion: A case of NCAA Division I head coaches. Am J Psychol.

[35] Vinberg M., Durbeej N., Rosendahl I. (2020). Gambling and gambling problem among elite athletes and their professional coaches: Findings from a Swedish total population survey of participants in four sports. Int Gambling Studies.

[36] Smith A., Torres-McGehee T., Monsma E., Gay J. (2018). Prevalence of eating disorder risk and body image perceptions of collegiate cheerleading coaches. J Sports Med Allied Health Sci.

[37] Purcell R., Gwyther K., Rice S.M. (2019). Mental health in elite athletes: Increased awareness requires an early intervention framework to respond to athlete needs. Sports Med Open.

[38] Purcell R., Henderson J., Tamminen K.A. (2023). Starting young to protect elite athletes’ mental health. Br J Sports Med.

[39] Davey C.G., McGorry P.D. (2019). Early intervention for depression in young people: A blind spot in mental health care. Lancet Psychiatr.

[40] McGorry P.D., Purcell R., Goldstone S., Amminger G.P. (2011). Age of onset and timing of treatment for mental and substance use disorders: Implications for preventive intervention strategies and models of care. Curr Opin Psychiatr.

[41] Correll C.U., Galling B., Pawar A. (2018). Comparison of early intervention services vs treatment as usual for early-phase psychosis: A systematic review, meta-analysis, and meta-regression. JAMA Psychiatr.

[42] Bertelsen M., Jeppesen P., Petersen L. (2008). Five-year follow-up of a randomized multicenter trial of intensive early intervention vs standard treatment for patients with a first episode of psychotic illness: The OPUS Trial. Arch Gen Psychiatr.

[43] Chanen A.M., Jackson H.J., McCutcheon L.K. (2008). Early intervention for adolescents with borderline personality disorder using cognitive analytic therapy: Randomised controlled trial. Br J Psychiatr.

[44] Malla A.K., Norman R.M.G., Joober R. (2005). First-episode psychosis, early intervention, and outcome: What have we learned?. Can J Psychiatr.

[45] Merry S.N., Hetrick S.E., Cox G.R., Brudevold-Iversen T., Bir J.J., McDowell H. (2011). Psychological and educational interventions for preventing depression in children and adolescents. Cochrane Database Syst Rev.

[46] Powell S.M., Fasczewski K.S., Stevens N. (2022). Pressure, stress, and coping: Exploring the professional demands of NCAA Division I coaching. J Sport Behav.

[47] Kaski S.S., Kinnunen U. (2021). Work-related ill- and well-being among Finnish sport coaches: Exploring the relationships between job demands, job resources, burnout and work engagement. Int J Sport Sci Coaching.

[48] Bentzen M., Lemyre P.-N., Kenttä G. (2016). Development of exhaustion for high-performance coaches in association with workload and motivation: A person-centered approach. Psychol Sport Exerc.

[49] Gouttebarge V., Bindra A., Blauwet C. (2021). International Olympic Committee (IOC) Sport Mental Health Assessment Tool 1 (SMHAT-1) and Sport Mental Health Recognition Tool 1 (SMHRT-1): ttowards better support of athletes’ mental health. Br J Sports Med.

[50] Rice S.M., Parker A.G., Mawren D. (2020). Preliminary psychometric validation of a brief screening tool for athlete mental health among male elite athletes: the Athlete Psychological Strain Questionnaire. Int J Sport Exerc Psychol.

[51] Rice S., Olive L., Gouttebarge V. (2020). Mental health screening: Severity and cut-off point sensitivity of the Athlete Psychological Strain Questionnaire in male and female elite athletes. BMJ Open Sport Exerc Med.

[52] Harris B.S., Ostrow A.C., Humphrey J.H. (2008). Sports and Athletics Developments.

[53] Schaffran P., Kleinert J., Altfeld S., Zepp C., Kallus K.W., Kellmann M. (2019). Early risk detection of burnout: Development of the burnout prevention questionnaire for coaches. Original research. Front Psychol.

[54] Lundkvist E., Gustafsson H., Gerber M., Lundqvist C., Ivarsson A., Madigan D.J. (2019). Commentary: Early risk detection of burnout: Development of the burnout prevention questionnaire for coaches. General commentary. Front Psychol.

[55] Bentzen M., Kenttä G., Richter A., Lemyre P.-N. (2020). Impact of job insecurity on psychological well- and ill-being among high performance coaches. Int J Environ Res Publ Health.

[56] Bentzen M., Kenttä G., Lemyre P.-N. (2020). Elite football coaches experiences and sensemaking about being fired: An interpretative phenomenological analysis. Int J Environ Res Publ Health.

[57] Weber B., Bos J., Clancy E.M., Menon R., Cross T., Hall K. (2022). Role of club doctors in the mental health management of Australian rules football players: A Delphi study. Br J Sports Med.

[58] Kenttä G., Mellalieu S., Roberts C.-M. (2016). Are career termination concerns only for athletes? A case study of the career termination of an elite female coach. Sport Psychol.

[59] Kelly C.M., Jorm A.F., Wright A. (2007). Improving mental health literacy as a strategy to facilitate early intervention for mental disorders. Med J Aust.

[60] Gorczynski P., Gibson K., Clarke N., Mensah T., Summers R. (2020). Examining mental health literacy, help-seeking behaviours, distress, and wellbeing in UK coaches. Eur Phys Educ Rev.

[61] Sullivan P., Murphy J., Blacker M. (2019). The level of mental health literacy among athletic staff in intercollegiate sport. J Clin Sport Psychol.

[62] Bissett J.E., Kroshus E., Hebard S. (2020). Determining the role of sport coaches in promoting athlete mental health: A narrative review and Delphi approach. BMJ Open Sport Exerc Med.

[63] Brown M., Deane F.P., Vella S.A., Liddle S.K. (2017). Parents views of the role of sports coaches as mental health gatekeepers for adolescent males. Int J Mental Health Promotion.

[64] Mazzer K.R., Rickwood D.J. (2009). Community gatekeepers' advice to young people to seek help from mental health professionals: Youth workers and sport coaches. Int J Mental Health Promotion.

[65] Castaldelli-Maia J.M., de Mello e Gallinaro J.G., Falcao R.S. (2019). Mental health symptoms and disorders in elite athletes: A systematic review on cultural influencers and barriers to athletes seeking treatment. Br J Sports Med.

[66] Sebbens J., Hassmén P., Crisp D., Wensley K. (2016). Mental health in sport (MHS): Improving the early intervention knowledge and confidence of elite sport staff. Front Psychol.

[67] Vella S.A., Swann C., Batterham M. (2021). An intervention for mental health literacy and resilience in organized sports. Med Sci Sports Exerc.

[68] Hägglund K., Kenttä G., Thelwell R., Wagstaff C.R.D. (2022). Mindful self-reflection to support sustainable high-performance coaching: A process evaluation of a novel method development in elite sport. J Appl Sport Psychol.

[69] Longshore K., Sachs M. (2015). Mindfulness training for coaches: A mixed-method exploratory study. J Clin Sport Psychol.

[70] Vella S.A., Mayland E., Schweickle M.J., Sutcliffe J.T., McEwan D., Swann C. (2022). Psychological safety in sport: A systematic review and concept analysis. Int Rev Sport Exerc Psychol.

[71] Edmondson A. (1999). Psychological safety and learning behavior in work teams. Admin Sci Q.

[72] Rice S., Walton C.C., Pilkington V. (2022). Psychological safety in elite sport settings: A psychometric study of the Sport Psychological Safety Inventory. BMJ Open Sport Exerc Med.

[73] Hjälm S., Kenttä G., Hassmén P., Gustafsson H. (2007). Burnout among elite soccer coaches. J Sport Behav.

[74] Baldock L., Cropley B., Mellalieu S.D., Neil R. (2022). A longitudinal examination of stress and mental ill-/well-being in elite football coaches. Sport Psychol.

[75] Gulliver A., Griffiths K.M., Christensen H. (2012). Barriers and facilitators to mental health help-seeking for young elite athletes: A qualitative study. BMC Psychiatr.

[76] Ojio Y., Matsunaga A., Yamaguchi S. (2021). Association of mental health help-seeking with mental health-related knowledge and stigma in Japan Rugby Top League players. PLOS ONE.

[77] Poucher Z.A., Tamminen K.A., Kerr G., Cairney J. (2021). A commentary on mental health research in elite sport. J Appl Sport Psychol.

[78] Rice S., Butterworth M., Clements M. (2020). Development and implementation of the National Mental Health Referral Network for Elite Athletes: A case study of the Australian Institute of Sport. Case Studies Sport Exerc Psychol.

[79] Tozetto A.B., Silva W.R., Carvalho H.M. (2019). Coach turnover in top professional Brazilian football championship: A multilevel survival analysis. Front Psychol.

[80] Ardern C.L. (2015). Anterior cruciate ligament reconstruction—Not exactly a one-way ticket back to the preinjury level: A review of contextual factors affecting return to sport after surgery. Sports Health.

[81] Olusoga P., Butt J., Maynard I., Hays K. (2010). Stress and coping: A study of world class coaches. J Appl Sport Psychol.

[82] Hassmén P., Kenttä G., Hjälm S., Lundkvist E., Gustafsson H. (2019). Burnout symptoms and recovery processes in eight elite soccer coaches over 10 years. Int J Sports Sci Coaching.

[83] Kellmann M., Altfeld S., Mallett C.J. (2016). Recovery–stress imbalance in Australian Football League coaches: A pilot longitudinal study. Int J Sport Exerc Psychol.

[84] de Sousa Pinheiro G., Túlio de Mello M., Gustavo dos Santos F., Fiorese L., Teoldo da Costa V. (2021). Analysis of stress level and recovery of formative football coaches. Case studies. Retos.

[85] Currie A., Purcell R. (2021). Sport psychiatry and its research agenda. Psychiatr Clin North Am.

[86] Walton C.C., Gwyther K., Gao C.X., Purcell R., Rice S. (2022). Evidence of gender imbalance across samples in sport and exercise psychology. Int Rev Sport Exerc Psychol.

[87] Serpell B.G., Harrison D., Dower R., Cook C.J. (2023). The under representation of women coaches in high-performance sport. Int J Sports Sci Coaching.

[88] Kenttä G., Bentzen M., Dieffenbach K., Olusoga P. (2020). Challenges experienced by women high-performance coaches: Sustainability in the profession. Int Sport Coaching J.

[89] O'Connor M., Casey L. (2015). The Mental Health Literacy Scale (MHLS): A new scale-based measure of mental health literacy. Psychiatr Res.

[90] Walton C.C., Osborne M.S., Gilbert P., Kirby J. (2022). Nurturing self-compassionate performers. Aust Psychol.

